# Barriers to publishing early phase clinical trials: the oncologists’ perspective

**DOI:** 10.1093/oncolo/oyaf042

**Published:** 2025-04-24

**Authors:** Merel J J Lucassen, Pedro Bergmann, Olga Husson, Udai Banerji, Bristi Basu, Ignacio Melero, Emiliano Calvo, Philippe A Cassier, Alexander Drilon, Peter C Fong, Elena Garralda, Anthony M Joshua, Chia-Chi Lin, Juanita Lopez, Victor Moreno, Anna Minchom, Ruth Plummer, Sophie Postel-Vinay, Anna Spreafico, Toshio Shimizu, Timothy A Yap, Christina Yap, Johann S De Bono, Neeltje Steeghs

**Affiliations:** Department of Medical Oncology, The Netherlands Cancer Institute, Amsterdam 1066CX, The Netherlands; The Royal Marsden NHS Foundation Trust, London SW3 6JJ, United Kingdom; Department of Medical Oncology, The Netherlands Cancer Institute, Amsterdam 1066CX, The Netherlands; The Institute of Cancer Research, London SW7 3RP, United Kingdom; University of Cambridge and Cambridge University Hospitals NHS Foundation Trust, Cambridge CB2 1TN, United Kingdom; Clinica Universidad de Navarra, CIMA and CIBERONC, Pamplona 31008, Spain; Nuffeld Department of Medicine, University of Oxford, Oxford OX3 7BN, United Kingdom; START Madrid-CIOCC, Centro Integral Oncológico Clara Campal, Madrid 28050, Spain; Centre Léon Bérard, Lyon 69008, France; Memorial Sloan Kettering Cancer Center and Weill Cornell Medical Center, New York, NY 10065, United States; Auckland City Hospital and the University of Auckland, Auckland 1023, New Zealand; Val d’Hebron Institute of Oncology, Barcelona 08035, Spain; Kinghorn Cancer Centre, St Vincent’s University Hospital, Sydney NSW 2010, Australia; National Taiwan University Hospital, Taipei 10617, Taiwan; The Royal Marsden NHS Foundation Trust, London SW3 6JJ, United Kingdom; The Institute of Cancer Research, London SW7 3RP, United Kingdom; START Madrid-FJD, Hospital Universitario Fundacion Jimenez Diaz, Madrid 28040, Spain; The Royal Marsden NHS Foundation Trust, London SW3 6JJ, United Kingdom; The Institute of Cancer Research, London SW7 3RP, United Kingdom; Northern Centre for Cancer Care, Newcastle University, Newcastle upon Tyne NE7 7DN, United Kingdom; Gustave Roussy and Paris-Saclay University, Villejuif 94800, France; University College of London Cancer Institute, London WC1E 6DD, United Kingdom; Princess Margaret Cancer Centre, University Health Network, Toronto, ON M5G 2M9, Canada; Kansai Medical University Hospital, Osaka 573-1191, Japan; The University of Texas MD Anderson Cancer Center, Houston, TX 77030, United States; The Institute of Cancer Research, London SW7 3RP, United Kingdom; The Royal Marsden NHS Foundation Trust, London SW3 6JJ, United Kingdom; The Institute of Cancer Research, London SW7 3RP, United Kingdom; Department of Medical Oncology, The Netherlands Cancer Institute, Amsterdam 1066CX, The Netherlands; University Medical Center Utrecht, Utrecht 3584 CX, The Netherlands

**Keywords:** qualitative research, ethics, medical oncology, publication

## Abstract

**Introduction:**

Findings from early phase studies are not always placed in the public domain. This study aims to explore why many early phase clinical oncology studies are not published, as well as identify the potential barriers investigators encountered in the publication process.

**Methods:**

Semi-structured interviews were conducted among investigators with experience in early phase clinical oncology studies. Interviews were analyzed using reflexive thematic analysis.

**Results:**

Twenty-one investigators were interviewed. The majority worked in Europe (*n* = 13), while other investigators were based in North America (*n* = 4), Asia (*n* = 2) or Oceania (*n* = 2). We identified three reasons why investigators believed publishing early phase clinical trial results was important: (1) there is an ethical and moral responsibility; (2) there should be no loss of knowledge to society; and (3) there should be no waste of resources. Four main barriers in the publication process of early phase clinical trials were identified: (1) practical barriers (eg, an increased complexity of number of trials/trial sites), (2) insufficient resources (eg, money, time and human), (3) limited motivation (eg, limited intrinsic motivation of the investigator or limited prospect of return for the sponsor), and (4) inadequate collaboration (eg, different interests between industry partners and investigators). Finally, five major stakeholders were identified that can potentially contribute to improving the publication process: (1) journal editors, (2) sponsors, (3) investigators, (4) regulatory bodies, and (5) society. Investigator suggestions for improving this process, for each stakeholder, are presented.

**Conclusions:**

This study highlights the barriers experienced in publishing early phase clinical trials. Recognizing and acknowledging these barriers is crucial to devise effective strategies to improve the publishing and public sharing of early phase clinical trials.

**Implications for practice**

This qualitative analysis defines the barriers experienced by oncologist when publishing early phase clinical trials. Semi-structured interviews with 21 investigators were conducted and subsequently analyzed using reflexive thematic analysis. The main barriers are: (1) practical barriers, (2) insufficient resources, (3) limited motivation, and (4) inadequate collaboration. Stakeholders were identified who can help improve the publication process: editors, sponsors, investigators, regulatory bodies, and society. Acknowledging these barriers is essential for developing effective strategies to enhance the publication and public dissemination of early phase clinical trial results.

## Introduction

Since the development of the Declaration of Helsinki in 1964, which states in paragraph 36 “*Researchers, authors, sponsors, editors and publishers all have ethical obligations with regard to the publication and dissemination of the results of research*,” the importance of publishing clinical study results has found its way to clinical research guidelines and regulations.^1^ The practical application of paragraph 36 is reflected in regulations. For example, the European Medicines Agency (EMA), Food and Drug Administration (FDA), and Medicines and Healthcare products Regulatory Agency (MHRA) require a sponsor to publish a trial summary within 1 year after the end of a clinical trial. The FDA mandates under the Code of Federal Regulations 42CFR11.2(44) that results are to be published at Clinicaltrials.gov, the MHRA requires a publication of trial results on a trial registry platform such as Clinicaltrials.gov or the ISRCTN registry and the EMA manages the Clinical Trials Information System that replaced the EU Clinical Trials Register in January 2023. All clinical trials performed in the European Union under the Clinical Trials Regulations are to be registered within this system and require a trial summary within 1 year after study completion. Therefore, publishing results of clinical trials is often a regulatory requirement.

Multiple studies have shown that not all clinical trials are being published.^2-5^ As of 2022, 81% of trials in the EU Clinical Trials Register and as of March 2024, 77% of trials at Clinicaltrials.gov are compliant with their regulatory obligation to report the study results.^5,6^ However, these results are not a reliable reflection of the proportion of published phase I trials. While the ethical standards outlined in the Declaration of Helsinki are applicable to all clinical trials, there is a gap in the regulatory policies for phase I trials. The FDA Amendments Act exempts phase I trials from their registration and publication policy (as they are referred to as not an “Applicable Clinical Trial”) to align this policy with existing regulation and to protect the incentive to invest in innovative research.^7^ And the EMA relieves phase I trials from their policy to make the trial information publicly available to protect the legitimate interests of sponsors. It has been emphasized that these policies might merit review to improve the availability of knowledge pertaining to the early stages of drug development.^8-11^ This is particularly pertinent given the fact that only 49%-72% of phase I trials are published in a peer-reviewed format.^12-14^ Most phase I trials provided limited and/or insufficient information in an abstract format.^12,15,16^ Conversely, the early phases of drug development can provide insight into the mecahnisms of action, safety profiles of new compounds and/or influence the evolving drug development landscape.

Little is known about the reasons why these early phase clinical trials are not published and what obstacles are experienced. The aims of this study were to examine the motivations of investigators behind publishing early phase clinical trials in cancer patients and identify potential barriers they encounter when publishing early phase clinical trial results. Additionally, possible solutions to these barriers were explored.

## Methods

### Study design

A qualitative study was conducted using reflexive thematic analysis previously described by Braun and Clarke (2006).^17^ The focus was on identifying broad patterns across the data. The analysis was conducted from a critical realist perspective, assuming the existence of a pursuable reality while acknowledging that representation of this reality is characterized by factors such as the interviewee’s culture, language, and interest.^18^ This approach was chosen to understand the actions and ideas of the participant in the context of their work and challenges they experience.

### Procedure

Participants were recruited for the project via an email stating that in the experience of the 3 lead investigators (C.Y., J.B., N.S.) there is an increased sentiment that results of early phase clinical trials are not being shared with the public. The email asked if the recipient had similar experiences or not and if they were willing to talk about their experiences. The recipients were selected on the basis of their expertise and experience in early phase clinical trials and previous collaborations with the 3 lead investigators. Eligible participants were oncologists or methodologists with experience in early phase clinical trials, hereafter described as “investigators.” March 2023, a meeting was scheduled with the investigators to gain active engagement with the project, followed by 30-min interviews to collect in-depth experiences of all investigators. Data collection stopped when saturation was reached, next to the minimum predefined sample of investigators and when the qualitative researcher had a clear overview of the experiences of the investigators. Data saturation was defined as the point at which no new themes or codes emerge from the interviews.

Interviews were conducted between May 2023 and August 2023. A qualitative researcher (M.L.) conducted the semistructured in-depth interviews via video calls. An interview guide with open questions and probes was developed based on literature and the meeting with the investigators. This interview guide was drafted with experienced researchers (O.H., N.S.). The interview guide was pilot-tested on one researcher with experience in qualitative research and one medical doctor with experience in early phase clinical trials who were not associated with this study. Minor adjustments were done after the pilots and the pilot data was not included in the analysis. Supplementary Table S1 reflects the interview guide. Videos were auto-recorded, transcribed and lasted on average 27 min (range: 21-43 min). Notes and summaries were made to provide context for the analysis.

### Data analysis

The interviews were transcribed verbatim and the transcripts were analyzed by P.B. and M.L. using reflexive thematic analysis. Analysis started after the first interview. Each interview and analysis was used to reflect on the interview guide and adapt when necessary. Analysis started with open coding using NVivo v14. Reflexive thematic analysis involved familiarization with data, generating initial codes, searching for themes, reviewing themes, defining and naming themes, and producing the report. First 7 interviews were separately coded by M.L. and P.B. to identify and discuss different perspectives on the same data. Relevant excerpts of the interviews were semantically coded. Codes were discussed among M.L. and P.B. until agreed upon. After the seventh interview, M.L. and P.B. concluded a similar coding structure and extensive codebook. The remaining 14 interviews were coded by M.L. The codes were sorted by M.L., N.S., and O.H. into meaningful themes by using a dendritic structure of a central theme from which subthemes emerge. Themes and subthemes were mapped, revised, and refined to ensure a good fit with the data. The themes were actively created by the research team (M.L., O.H., N.S., C.Y., J.B.) through the interplay of the data, analytic process, and researchers subjectivity. All the data came to a plausible explanatory framework about the barriers that investigators experience when conducting early phase clinical trials. This general framework was checked for misinterpretations or gaps by the research team and investigators as active research partners, resulting in minor adjustments. The Guidance for Reporting Involvement of Patients and the Public checklist (GRIPP2-SF) in Supplementary Table S2 reflects the investigators involvement as research partners and coauthors of this manuscript.

## Results

In total, 26 investigators were invited to participate, of whom 16 participated in the meeting and 21 (81%) were interviewed. Three investigators did not respond and 2 investigators replied to be in agreement with the proposed sentiment in the email, but were not able to participate. Due to conceptual saturation after the 21 initially planned interviews, no additional investigators were invited.^19^ All investigators worked in a medical center and had experience in early phase clinical trials in oncology. The majority worked in Europe (*n* = 13), while other investigators were based in North America (*n* = 4), Asia (*n* = 2) or Oceania (*n* = 2). Twenty out of 21 investigators are medical oncologists, and one investigator is a trial methodologist and statistician.

**Figure 1.**
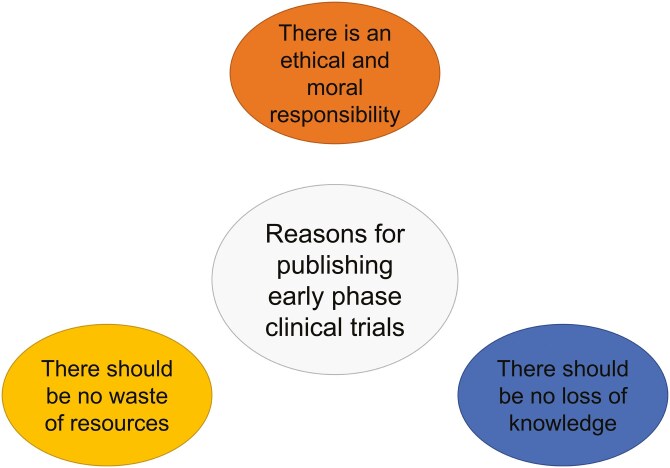
Reasons why investigators believe there is a need to publish early phase clinical trials.

Three predominant reasons were identified as to why early phase clinical trial results should be published: (1) there is a moral or ethical responsibility, (2) there should be no loss of knowledge, and (3) there should be no loss of resources such as money or time. The themes, codes, and illustrative quotes for the interpretation of the codes are presented in Figure 1 and in Supplementary Table S3. Investigators believe that publishing early phase clinical trials should be standard practice. They feel a moral responsibility and an obligation toward their patients to publish the results of early phase clinical trials. Investigators want to respect their own patient’s wishes, as the patient was exposed to potential toxicity and invasive procedures under the impression that their offerings might help science:

*“One of the motivations going into a phase one trial, it’s mentioned very regularly to me, is about contributing to the development of new drugs to treat cancer. You know, “if it’s not going to help me, it might help somebody else.” So therefore your* (investigator) *part of the deal is to make sure that their* (patients) *data isn’t lost and is used to try and help treat cancer patients in the future.”*

Secondly, investigators want to prevent harm to future patients if a trial is repeated. Twenty out of the 21 investigators named publishing of the results a moral or ethical responsibility.

In addition to the moral and ethical obligation, investigators view that the knowledge that is gained with a clinical trial should not be lost and resources should not be wasted. Investigators feel it as their duty to increase worldwide knowledge sharing in the scientific community and advance science. And they specify that clinical trials with compounds that are not being further developed should also be addressed as potential sources of important information. More generally, investigators view that consumption of resources such as money or time, without contributing to the body of knowledge, should be avoided.

### Barriers to publishing early phase clinical trials

**Figure 2.**
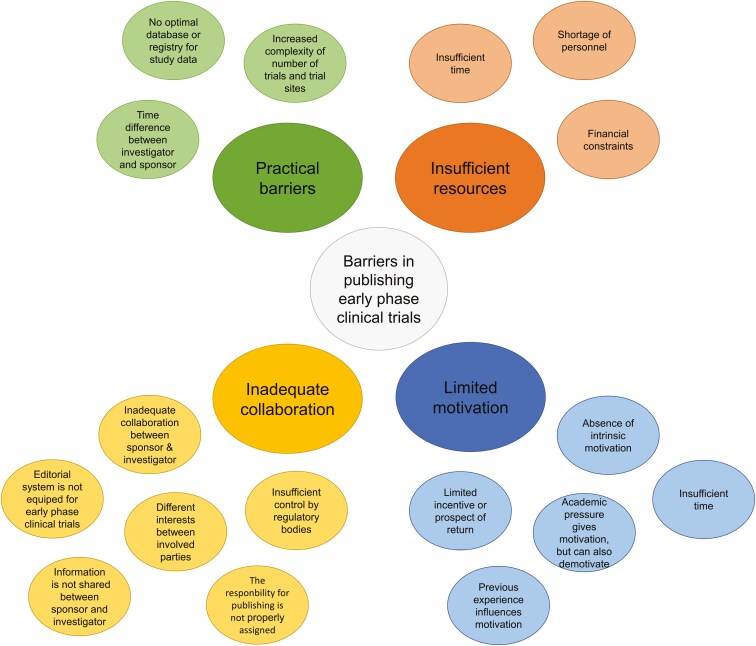
Barriers that investigators experience in publishing early phase clinical trials. Darker colors represent main themes and lighter colors represent subthemes.

Four themes were identified that reflect the barriers investigators experience in publishing early phase clinical trial results: (1) practical barriers, (2) insufficient resources, (3) limited motivation, and (4) inadequate collaboration. These collaborations involve different types of institutes; academic or industrial. Academic studies are most often referred to as studies that have a (academic) medical center as sponsor. Industrial studies are studies that involve a commercial party as sponsor, for example, a pharmaceutical company or a biotech company. A visual overview of the themes and subthemes is presented in Figure 2. Supplementary Table S4 gives an overview of the themes, subthemes, and codes along with illustrative quotes for the interpretation of the codes.

#### Theme 1. Practical barriers

When investigators want to publish early phase clinical trial results, they have practical barriers that they sometimes have to overcome. Current database registries, such as ClinicalTrials.gov, are suboptimal to report trial results (subtheme 1.1). It is difficult to upload large PDF files and consequently the information that is in the database is reduced to only the most essential information in the eye of the submitting investigator. Therefore, investigators find that they have limited options if trial results are difficult to publish in a peer-reviewed format, for example, due to their limited efficacy results or suboptimal trial design.

Secondly, investigators highlight that more complex trials, such as umbrella or basket trial designs, and more trial sites per study also complicate the publication process (subtheme 1.2). With such studies, it is often more difficult to pinpoint when to publish trial results. For example, one investigator stated:

*“They open 30 sites, so none of the PI’s* (principal investigators) *really (…) own it. So if you do a 3 center study, you’re passionate about the drug, you get to own it, even if it’s for failure. You know multiple of treated patients and multiple dose levels. Here they open to expansion in 30-40 sites. So there’s nobody really in charge of spearheading.”*

Especially for the trial arms that have negative safety or efficacy results and are not being further investigated, the publication of results has little attention. Additionally, investigators feel less of a moral obligation to ensure publishing when they have limited patients involved in the trial.

Some investigators that are established in non-EU or non-USA time zones also experience time difference as a practical barrier (subtheme 1.3). They highlight that they experience reluctance with industry partners to work with non-EU or non-USA centers due to the practical issues that arise from different time zones (eg, attending a close-out visit to discuss the publication process).

#### Theme 2. Insufficient resources

Investigators experience a restriction in resources when publishing early phase clinical trials that involve compounds that are not being pursued for future research. These insufficient resources are referred to as financial constraints, shortage of personnel and limited time. Regularly, they are described as intertwined in relation to each other. The financial constraints (subtheme 2.1) that are subsequent to a compound that is not being further developed often result in reallocation of the current staff to another project in an industry setting (subtheme 2.2). In the academic setting, financial constraints (subtheme 2.1) and shortage of personnel in clinical care (subtheme 2.2) result in the inability to free investigators from daily clinical duties for clinical research (subtheme 2.3):

*“When the drug is no longer in development (…) most companies are reluctant to get it published. It is not because they do not want this result to be public. I think it is simply they have a new focus. And they need to put effort in another direction. (…) And even the study team has dissolved. It is no longer there. The company relocated them to another project. I think it is very hard to publish then.”*

Clinical care is described as time-consuming and often more urgent in day-to-day practice than publishing a trial. Additionally, in the academic environment, there is often a transient workforce that leaves a gap in personnel for a trial when their contract ends.

#### Theme 3. Limited motivation

Motivation to publish trial results is a factor that investigators experience as limiting due to their own experiences or due to external influences of collaborative partners. The intrinsic motivation of the investigator is especially limited in regard to trials that involve compounds that are not being pursued and/or show limited efficacy (subtheme 3.1):

*“The drugs that are doing well usually there is a determination to publish them. The drugs that are not doing that well nobody cares and nobody is really determined to spend time on that. You know the difference is that the ones that are working, you get access to higher impact factor journals.”*

The academic environment is described as motivating for the investigators, as a publication is often necessary for academic career advancement. However, an investigator might want the trial results published in a journal that has the most beneficial effect on their career and therefore has to invest more time and resources, which are both scarce (subthemes 3.2, 3.3, 3.4).

Additionally, the investigators experience limited motivation of the pharmaceutical industry to publish the results in a peer-reviewed article when the compound is not being pursued. This is motivated by a reallocating of resources when there is limited of prospect of return (subtheme 3.5).

#### Theme 4. Inadequate collaboration

Investigators have encountered obstacles in their collaboration with sponsors, regulatory bodies, and the editorial system. They describe a sense of limited support when sponsors are hesitant to invest in the publication of early phase clinical trial results without explanation of the sponsor or due to limitations in the sponsors’ resources, logistics, and/or finances. In such cases, the investigators tend to perceive the regulatory bodies as being unable to provide assistance, citing the lack of existence, and/or enforcement of publication policies. Furthermore, investigators perceive their own capacity to enforce contractual obligations with sponsors to publish the results to be constrained due to limitations in their knowledge and resources (subtheme 4.1):

*“These things* (publishing commitments) *are all usually in the contract. But these are never, ever upheld. You can’t bring them to court if they’re not publishing. Because there are usually issues why they’re not publishing.”*

Also, when an investigator and sponsor have different interests, there are obstacles in the cooperation to get the clinical trial data published (subthemes 4.2, 4.3):

Investigator giving an example of a study that is not published yet *- “We’ve had several calls, they’re just not interested. They say, “We would love to, but we need to concentrate on the next asset and we don’t have the resource to look into”. (…) You know it has to be in agreement that this is going to be published because otherwise why would we put our patients through it.”*

These barriers can be due to different timelines of the parties involved in the clinical trial, the (un)available resources, the reluctance of a partner to share clinical trial data (subtheme 4.4), or the improper assignment of the responsible party (subtheme 4.5). For example, if there is an unclear authorship determination, no close-out visit or there is no clear lead investigator, the start of a manuscript writing phase can be difficult to determine.

Lastly, investigators find the editorial system not properly equipped for early phase clinical trials (subtheme 4.6). Studies that involve compounds that are not being further developed due to no preliminary efficacy or pharmacodynamics results, or suboptimal trial designs, are experienced as more difficult to publish in a peer-reviewed format.

### Possible solutions that might improve publishing early phase clinical trials

Investigators were prompted to provide possible solutions that might contribute to improving the publication process. The main objective was to identify possible barriers that had not yet surfaced in the interview. It was not an objective to discuss the details, feasibility or implementation of these solutions.

**Figure 3.**
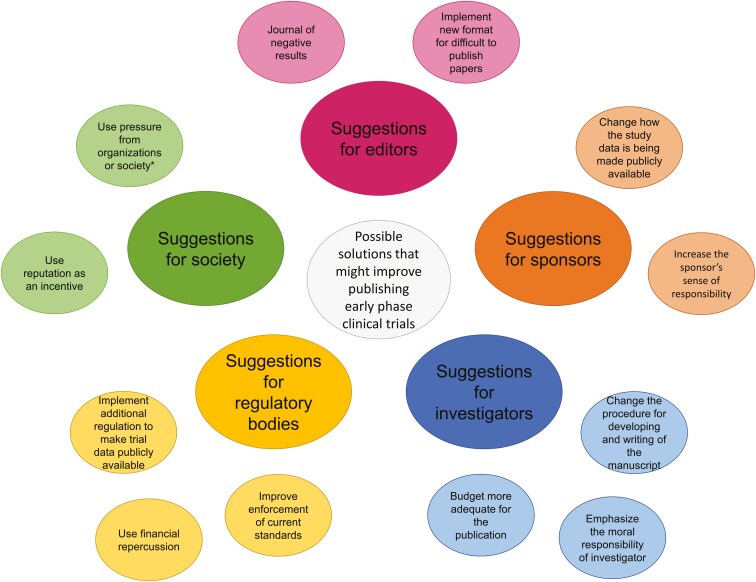
Identified possible solutions to improve the publication process of early phase clinical trials. Darker colors represent main themes and lighter colors represent subthemes. *Examples of organizations or society are ethics committees, patient advocacy groups, or the academic community.

Five major stakeholders were identified that can potentially contribute to improving the publication process: (1) editors, (2) sponsors, (3) investigators, (4) regulatory bodies, and (5) society. A visual overview of the stakeholders represented as themes and an exploration of possible solutions in the eye of the investigator are represented as subthemes in Figure 3. Supplementary Table S5 gives an overview of the possible solutions along with illustrative quotes for the interpretation of the codes.

#### Stakeholder A. Editors

Investigators experience barriers in the publication process when attempting to disseminate findings from early phase clinical trials. This is particularly evident in the case of a manuscript involving an early phase clinical trial with a compound that is not being pursued further. In response, some investigators are seeking a new format that is both of high quality and less time-consuming and that is welcomed by journal editors. This would make the publication process more accessible for such manuscripts (subtheme A.1):

*“Journals also want highly cited publications, so no negative studies. They are going to be read, but not going to be cited so much. So why publish this. But perhaps if all journals had a couple of spots in each journal (…) for a section of drugs that are not gonna go. (…) Then everybody in the metric it levels* [sic]. *Because if only one does it, they are publishing a lot and it is bad for the metrics.”*

Another exploration of interest might be a journal of negative results (subtheme A.2); when a compound is not being further developed, the trial can still be published and the information is not lost to the public domain.

#### Stakeholder B. Sponsors

Collaborations between investigator and sponsor can be more effective. Investigators suggest alternative platforms and formats to make the study data available in the public domain if publishing in a peer-reviewed format is suboptimal. Examples of a registry of completed trials, open-access data, or to publish the regulatory study reports (subtheme B.1) were given. And secondly, responsibility and accountability of investigator, sponsor, and other parties involved in the clinical trial, can be explored (subthemes B.2, C.1).

*“I just think there just needs to be responsibility and accountability on the part of the trial sponsor, who are the IND* (Investigational New Drug) *holders, to ultimately publish.”*

#### Stakeholder C. Investigators

Investigators view their own role in improving the publication process with practical and ethical ideas. Practical suggestions such as planning the publication at study start, to invest in a close-out visit, to ask junior doctors to help with manuscript development (subtheme C.2) or to budget more adequate for the publication process (subtheme C.3), were prompted.

#### Stakeholder D. Regulatory bodies

The investigators tend to see a role for the regulatory authorities in making mandatory requirements for publishing of clinical trial results (subthemes D.1, D.2) and enforcing the current standards (subthemes D.1, D.3).

*“From the regulatory perspective, if the health authorities make this mandatory, like they say, “well if you are not doing this, you’re not fulfilling your obligations from the health authority perspective”. And then, maybe when they’re presenting a new study to the regulatory agencies, they say, “no, we cannot evaluate your new study because you have pending issues from the prior one”. So this is a way to make it mandatory because contracts cannot.”*

#### Stakeholder E. Society

Different stakeholders that are not directly involved in the publication process are seen as parties that might have influence on the stakeholders involved. For example, societies like ESMO/ASCO, patient advocacy groups, ethics committees or the academic community (subthemes E.1, E.2).

*“Maybe we as an academic community have to rise up. And demand of the regulators to make this happen.”*

## Discussion

This study describes the barriers that investigators experience when publishing early phase clinical trials. We identified 4 main barriers that impact the publishing process; practical barriers, insufficient resources, limited motivation, and inadequate collaboration. Most of these barriers are only experienced by investigators when trying to publish a “negative study,” and not apply to “positive studies.” There is no true definition of a positive or negative study. In general, a “negative study” can be referenced to when talking about a study with low response rates, high toxicity and/or does not result in further development of the study drug. The barriers are more often related to “negative studies,” which include the majority of early phase studies as only approximately 10% of compounds tested in oncology phase I studies make it to marketing authorization.^20-22^

It may be that these barriers are interconnected. Especially “insufficient resources,” “limited motivation” and “inadequate collaboration” were frequently described as interacting factors in the publishing process. Inadequate collaboration between a sponsor and investigator is often related to financial constraints and limited human resources. Especially when a sponsor or research institute is unable or unwilling to bear the personnel costs and/or other financial burdens for a compound from which no return is expected, the collaboration between sponsor and investigator to disseminate the results is more challenging.^23,24^ As a result of these limitations, there seems to be a gap in the feeling of responsibility to publish the early phase trial results. Most individual investigators feel that the responsibility to publish the results is shared between the sponsor and investigator, but often perceive their individual possibility to intervene in the publishing process as limited. Therefore, possible solutions to the experienced barriers should be sought together with clear definition of the responsible stakeholders.

It is notable that most barriers encountered in the publication of early phase clinical trials were experienced by investigators in the context of a “negative study.” Conversely, the main rationale for the exemption of phase I clinical trials from registration and publication policies was the potential commercial interests. In light of this, a review of the regulatory policy may be warranted. Commercial confidentiality of new compounds may represent a relatively minor factor in the nonpublication of early phase clinical trial results. While stock performance may still be influenced by trial results, it should be reassessed if that outweighs the ethical responsibility to the patients and the potential loss of knowledge and resources.^25,26^

This feeling of responsibility is also seen in relation to the increased complexity of number of trials and trial sites. In 2021, the FDA announced its emphasis on dose optimization for oncology drugs with Project Optimus and in 2023, the Methodology for the Development of Innovative Cancer Therapies Taskforce brought a practical guide on the design and conduct of phase I trials of anticancer drugs. The potential benefits of a revision of the standard oncology phase I designs include a reduced morbidity and mortality for patients, reduced costs due to adverse effects and potentially faster new drug approvals. However, this optimization in methodologies can induce an increase in complexity of the trials.^27-29^ With complex trial designs, the duration of the entire trial is typically longer. Unless a publication plan is clearly outlined upfront, it is often unclear whether the results of the dose-escalation component should be published first or after completion of the entire trial, which could take considerably longer if the design permits adding additional dose expansion cohorts. The SPIRIT-DEFINE guidelines for early phase clinical trials addresses this by adding a new dissemination policy for early phase clinical trial protocols. This new policy introduces sharing of results while the trial is still ongoing, in addition to the pre-existing dissemination policy guideline to communicate trial results to the public.^30^ Our results highlight another challenge, as the feeling of responsibility of the individual investigator in regard to publishing the trial results is reduced with more complex trials.

The quality of the reporting of early phase trial results was no endpoint for this qualitative study. Nevertheless, some investigators commented on the reporting quality of early phase clinical trials, yet there seems to be no clear consensus on this issue. Some investigators proposed that deviating from the conventional peer-reviewed manuscript format for reporting early phase clinical trials may offer a potential solution to improve the publication process of “negative studies.” Alternatively, others emphasized the value of peer review as the golden standard. Suggested alternatives for the dissemination of results include the use of an easily accessible open-access data repository, a standardized short format for publication or the publication of non-peer-reviewed manuscripts. These solutions should be carefully reviewed by an expert panel for their added benefit and feasibility. The recently published CONSORT-DEFINE reporting guidelines set an internationally recognized standard for reporting of early phase clinical trial manuscripts.^31^

This study includes some suggestions on how to improve the publishing process. It was not an objective to discuss the details, feasibility or implementation of these solutions. Notably, some of the proposed solutions are not novel, but reflect standards that are already established although arguably not broadly practiced. For example, a number of journals have recognized barriers to publishing trials with negative results and have committed to including a greater number of studies with negative results, sometimes in a special format.^32-35^ Investigators were prompted to give solutions but not questioned about the feasibility of the solutions or their opinion on solutions that other investigators brought in. Consequently, these solutions should be interpreted only as possible suggestions. We specified suggestions per stakeholder involved in the publishing process, as the solutions can be discussed in a multidisciplinary environment. We are aware of the political component of the discussion about possible solutions. The influence of each stakeholder on each barrier should be explored, and thereafter their involvement for finding a solution can be addressed. With all stakeholders involved, subjects such as responsibility and/or accountability can be addressed as well.

The results of this study should be interpreted in light of some limitations. The selection procedure of investigators induced a selection bias. A practical inclusion method was opted to include investigators who had experience with barriers when publishing early phase clinical trials and the reflective thematic analysis approach ensured that the experience of one investigator is limited on the total results. Secondly, the selection method resulted in no inclusion of other stakeholders, who may have different opinions about publishing negative results (eg, about sharing of information given potentially commercially sensitive information). Future initiatives should take these differences between stakeholders into account. Thirdly, investigators from North America and Europe were more often represented than investigators from other continents. Different continents experience some differences in barriers. This is, for example, reflected in the practical barrier regarding time difference for conference calls. However, the United States and Europe account for the majority of clinical trials in the field of oncology. The International Clinical Trials Registry Platform accounts 55% of all clinical trials in the field of oncology to the United States and 29% to Europe.^36^ Lastly, the data of this study is based on single interviews which could limit the results. Investigators were encouraged to get in touch with the researcher if they recalled experiences or barriers that they did not discuss during the interview. Interviews, focus groups or discussion panels with a more diverse group, including all stakeholders, can shed more light on less common barriers and the influence that each stakeholder has in the publication process. This study was reported using the Standards for Reporting Qualitative Research (Supplementary Table S6).

This study provides the first step to improving the publishing process by uncovering the barriers faced by oncologists and methodologists in publishing early phase clinical trials. Recognizing and acknowledging these barriers is essential for developing effective strategies to enhance the dissemination and public sharing of early phase clinical trial results. Future initiatives could involve convening multidisciplinary stakeholders to identify impactful and feasible strategies for improving publication of early phase clinical trial results. This may entail fostering collaboration to establish upfront publication plans and adopting reporting guidelines, advocating for policy changes to address systemic barriers, and implementing education initiatives to raise awareness and improve research quality. By implementing these potential influential strategies, we can drive toward a more transparent landscape for publishing early phase clinical trial results.

## Supplementary Material

oyaf042_suppl_Supplementary_Tables_S1-S6

## Data Availability

The qualitative data generated and/or analyzed during this study are available from the corresponding author upon request. Types of data available are de-identified interview transcripts and thematic coding frameworks. The data will be provided in text format. No individual participant data will be shared and data will be shared in a manner that ensures the confidentiality and privacy of study participants. As such, any data containing personal or sensitive information has been de-identified. Direct quotes that could potentially lead to the identification of participants have been modified to ensure anonymity.
