# Supplementary material for: Barriers to publishing early phase clinical trials: the oncologists’ perspective
Source: Oncologist. 2025 Apr 24;30(4):oyaf042. doi: 10.1093/oncolo/oyaf042 (PMC12019228; doi:10.1093/oncolo/oyaf042)
Supplement: oyaf042_suppl_Supplementary_Tables_S1-S6 [file oyaf042_suppl_supplementary_tables_s1-s6.docx]

# Barriers to publishing early phase clinical trials: The oncologists’ perspective

## Supplementary Tables

Table S1. Interview guide

Table S2. GRIPP2-SF

Table S3. Reasons why investigators believe there is a need to publish early phase clinical trials

Table S4. Barriers that investigators experience in publishing early phase clinical trials

Table S5. Identified possible solutions to improve the publication of early phase clinical trials

Table S6. SRQR checklist

## **Table S1. Interview guide**

| **Essential question** | **Probes** |
| --- | --- |
| Why do you think it is important to publish  early phase trial data? | |
| How do you, in general, experience publishing phase I/II trials? | Have you encountered any problems trying to bring study results in the public domain?  Has publicizing phase I trials changed over the years?  In a collaboration, how do you discuss publishing? |
| In your ideal world, how would the publication of early phase clinical trials be improved? | What would your role be?  What information should be minimally published per trial?  What is included in a publication in the ideal world?  How should that information be published?  Who should have main responsibility for publishing? |

## **Table S2. GRIPP-2 SF Checklist**

| Section and topic | Item | Reported on page No |
| --- | --- | --- |
| 1: Aim Report the aim of the study | The aim of this study was to examine the potential barriers that investigators experience when publishing early phase clinical trials. | 2 |
| 2: Methods  Provide a clear description of the methods used for PPI in the study | 21 investigators were actively involved as research partners in this study. The investigators were PI’s or trial methodologists of early phase clinical trials and were invited to contribute as research partner. The recipients were selected on the basis of their expertise and experience in early phase clinical trials, and previous collaborations with the three lead investigators. They were invited via email and asked to participate in a focus group, called the ‘kick-off’ meeting. The results of this kick-off meeting gave input for the interview guide. At this meeting, the research partners were invited to contribute in the interview study. | 2-3 |
| 3: Study results  Report the results of PPI in the study, including both positive and negative outcomes | The investigators contributed to the study is several ways, including:   - Being a participant in the kick-off which resulted in a draft of the first interview guide - Checking for correct interpretation of the results - Checking the general framework for misinterpretation or gaps in logic - Editing and being co-author of the paper | 4-8 |
| 4: Discussion and conclusions  Comment on the extent to which PPI influenced the study overall. Describe positive and negative effects | Collaboration with the investigators as research partners provides a more appropriate, correct and relevant translation of their experiences and suggestions. Adjustments and prioritization of interview questions for the interview guide resulted in an improved methodology. After their review of the results, some themes and sub-themes were renamed to more appropriate names to reflect their correct interpretation. And the review of the general framework gave a more robust conclusion. | 8-9 |
| 5: Reflections/critical perspective  Comment critically on the study, reflecting on the things that went well and those that did not, so others can learn from this experience | This study engaged with a wide range of research partners, encompassing varying levels of experience, multiple continents and diverse types of clinical centers where the research partners conducted their early phase clinical oncology trials. All research partners demonstrated a high level of commitment and contributed to the study’s design in multiple ways (see sections 2, 3 and 4). Inclusion of a random sample of research partners would have ensured a less biased population of research partners, had the research team opted for this approach instead of selecting research partners in a practical matter. Furthermore, defining the themes and optimising the readability of the quotes were experienced as a challenging process by the research theme. A theoretical approach for defining the themes, as defined by Braun and Clarke (2006), could result in a more efficient process. [1] |  |

**References**

1 Braun V and Clarke V. Using thematic analysis in psychology. Qualitative research in psychology 2006;3:77-101.

## **Table S3. Reasons why investigators believe there is a need to publish early phase clinical trials**

| **Theme** | **Code** | **Quote** |
| --- | --- | --- |
| There is an ethical and moral responsibility | There is a moral obligation towards patients to publish | *“One of the motivations going into a phase one trial, it's mentioned very regularly to me, is about contributing to the development of new drugs to treat cancer. You know, ‘if it's not going to help me, it might help somebody else’. So therefore your* (investigator) *part of the deal is to make sure that their* (patients) *data isn't lost and is used to try and help treat cancer patients in the future.”* |
|  | Investigators feel an intrinsic moral responsibility to publish | *“By not publishing, it* (the trial) *actually does not mean anything. I think it is unethical not to publish.”* |
|  | Publishing ensures that investigators are transparent | *“Drug development is also a field where (…) people are perhaps a bit suspicious (…) So I think publishing is also transparency with all the stakeholders.”* |
|  | Publishing increases safety for same class toxicity | *“If we didn’t report dangers or toxicities and the same mistake happens with the same mechanism of action drug I think we are guilty. I would say we have a moral medical obligation to do that.”* |
|  | Society invests in expensive studies | *“I think it is our responsibility (…) for society as a whole. These are expensive studies. There are learnings to be gained.”* |
|  | Recognition for the work that the people involved in the trial did | *“It's important for everybody that's working on a study. Nurses, physicians, everybody. (...) So it's notable what they are doing and that they don't do their work without any results. The commitment is shown in results.”* |
| There should be no loss of knowledge | Trials with negative efficacy results should also be common knowledge and are equally important | *“Even if a drug failed (…) the negative experience is still important because it might tell us that biologically the tumors that were treated may not have responded for particular reasons. Also, it might tell us about how to design future drugs.”* |
|  | Data sharing stimulates new ideas and collaborations | *“It also stimulates new ideas, (…) when there's data sharing I think it can lead to increased collaboration. They may be doing something else, (…) for example when it's a small phase one in a small country, it doesn't necessarily mean that the phase one trial can’t reach other researchers across the globe who don't necessarily do a phase one trial, but they are interested in either perhaps the preclinical component.”* |
|  | Publishing increases safety for same class toxicity | *“It's really important for other drugs from the same class (…) to get the information from the first compound so that we can basically build on successes and failures”* |
|  | Publishing increases world-wide knowledge sharing in the scientific community and advances science | *“Because the data is interesting, because we want to share new information about new drugs and want to improve drugs in the future.”  “It is part of our job and duties as a scientist to share all information we can share with the community; medical and scientific.”*  *“To share the new knowledge is always good for the main goal of drug development, for our cancer patients.”* |
| There should be no waste of resources | Publishing prevents repetition of similar trials and waste of resources | *“By not publishing it, someone else might have to replicate it doing almost exactly the same thing. That is a huge waste* *of resources (…) also patients contributed significantly and thinking that their results and contribution will mean something. By not publishing it does actually not mean anything.”*  *“Even if the results are negative, the safety data is really important and it stops somebody else doing the same thing. Because what you're actually doing is potentially exposing patients to harm.”*  *“From an economical perspective, if we do share, if we do publish the data and that can lead also on a longer run to save some money, save some funding right, because you're not going to replicate.”* |

## **Table S4. Barriers that investigators experience in publishing early phase clinical trials**

| **Theme** | **Subtheme** | **Codes** | **Quote** |
| --- | --- | --- | --- |
| 1. Practical barriers | 1.1 No optimal databased registry for study data | Clinicaltrials.gov as data registry is suboptimal | *“Have you ever submitted anything to clinicaltrials.gov? It's very difficult to submit there and they're certainly not prepared for a large PDF file.”* |
|  | 1.2 Increased complexity of number of trials and trial sites | If there is no clear lead with a multicenter study, no one has the responsibility to push the industry partner for publishing | *“They open 30 sites, so none of the PI's* (principal investigators) *really (..) own it. So if you do a 3 center study, you're passionate about the drug, you get to own it, even if it's for failure. You know multiple of treated patients and multiple dose levels. Here they open to expansion in 30-40 sites. So there's nobody really in charge of spearheading.”* |
|  |  | Trials are more complex | *“The studies have changed, they’ve become more complex, they’ve become larger. (…) They are becoming Phase one-two, almost three. So they're getting more complex. So you never know when you want to say, OK, let's stop here. This is the data.”* |
|  | 1.3 Time difference between investigator and sponsor | The collaborations with non-EU/USA time-zones are more difficult due to time-difference | *“We don’t get a lot of offers, (…) nobody wants to come halfway across the world. I think they should. But it is really difficult.”* |
| 2. Insufficient resources | 2.1 Financial constraints | There is a lack of resources in studies sponsored by academic centers | *“There's definitely challenge when you're trying to do these studies (academic studies) off the back of a shoestring budget. Most of those studies are drug repurposing studies, the academic phase one studies or drug combination studies. It's relatively rare that an academic gets the first-in-class first-in-human molecule that has come out of their lab. Because that takes millions of dollars of investment. So mostly, they're other type of studies and it's important to get the information out, but it takes time and effort.”* |
|  |  | A lack of resources prevents publishing | *“If a trial is negative, there’s rapidly removed resource and funding. So it's a money issue (…) you take away the resource from it because you don’t want to waste money in publishing it.”*  *“So it's a resource commitment to the end of the study, which I don't think is often (…) enough.”* |
|  |  | Pharmaceutical sponsored trials have more resources to publish | *“I think the pharma trials, if they do get published, they get published a lot more faster* (than academic trials)*. Which is great. Obviously it needs somebody to get this* (publishing) *accomplished. But when they* (pharmaceutical company sponsored trials) *get published it is always resources that are pumped in there to get it published.”* |
|  |  | Small biotech companies don’t want to put resources on drugs that don’t work | *“For biotechs, it's definitely more complicated. Because they have less money, they have to have priorities. And they have timelines which are much shorter. So if they decide to kill a drug the impact is much higher than for a big pharma.”* |
|  | 2.2 Shortage of personnel | There is a lack of resources in academic studies | *“Sometimes there are people working on those studies, PhD students, and they end their project. And then it's never published. And there is not enough time for anybody to or but PI to take over. And it's not interesting, of course, for new PhD students.”* |
|  |  | A lack of resources prevents publishing | *“It takes too long to complete the trial. And by the time you have completed the trial, a lot of times in the academic world, you have the staff that started working on it no longer be the staff that finished it. So in a sense it takes much longer than you have funding for. You have very limited resources to actually continue to publish the results.“* |
|  |  | Contractual obligations cannot be enforced | *“So there are limited resources and this goes back to this business of sponsors saying “yes, it's coming. Yes, we will be writing it.” And you're two years past and they are batting it back and saying “yes, it will be coming.” How many resources can regulatory bodies put in to push it at that stage?”* |
|  |  | Study team dissolves due to new focus study team | *“When the drug is no longer in development (…) most companies are reluctant to get it published. It is not because they do not want this result to be public. I think it is simply they have a new focus. And they need to put effort in another direction. (…) And even the study team has dissolved. It is no longer there. The company relocated them to another project. I think it is very hard to publish then.”* |
|  | 2.1 Insufficient time | Publishing is time-consuming | *“Everything takes much longer to complete. By the time you have finished this, it is so much later. You have less resources.”* |
| 3.Lack of motivation | 3.1 Absence of intrinsic motivation | Not publishing is due to laziness | *“Even studies where you have minimal clinical activity. If you draw something interesting out of the prick of the translational data, your paper is going to be published in a reasonably big journal. I mean; it's always worth it. It's just that they're lazy. I think the issue is they are lazy.”* |
|  |  | Less enthusiasm to publish negative studies | *“If you know you are going to be doing an effort and the journal you are going to be publishing in is not really high impact factor (…) you de-prioritise that.”*  *“The drugs that are doing well usually there is a determination to publish them. The drugs that are not doing that well nobody cares and nobody is really determined to spend time on that. You know the difference is that the ones that are working, you get access to higher impact factor journals.”*  *“If you can't get into a high impact journal, most people lose interest.”*  *“For whatever reason, they've moved on to version two or they've moved on to more potent combination. You know, why bother?”* |
|  |  | It is a lot of effort to publish all trial data, sometimes it is easier to publish an abstract | *“We could finish this study three months ago, and we’re waiting for them to come back to us. Maybe they'll put an abstract together for ASCO/ESMO and that's it. (…) Anyone can write a quick abstract. But when we've actually published the data, that is a lot more effort.“* |
|  |  | Investigator being no chief investigator or lead center, feels less responsible to ensure publishing | *“I mean personally I don't contact the companies very often for with this regard because I don't feel super legitimate. I'm not the main recruiter and (…) I'm kind of an intermediate in the in the authorship list. So I don't feel super legitimate to contact the companies. And also I'm afraid that this has been discussed with the PI 's or the coordinating investigators or the lead authors and everyone has agreed.”* |
|  |  | Publication depends on willingness of investigator to invest to publish data | *“For this particular example, there was the end of study. They actually had a kind of follow-up TC* (telephone call) *to pull the data together, including the PD* (pharmacodynamic) *data. And we now are one year on, one and a half years on. And where's the draft manuscript? It's stuck. There are other companies, usually smaller biotech, where they don't have resource. Then, if the investigator has capacity, you can then say “okay well give me the CSR* (clinical study report)*, I'll (…) write it.”* |
|  | 3.2 Academic pressure gives motivation, but can also demotivate | There is a misbalance between importance to publish in high impact journals for academic career advancement versus the number and types of journals to publish negative trials | *“Most people will agree that the aspect of publication that wears you down is that you aim high and then you get rejection. And then you have to reform and come to the next paper down the impact factor ladder and down and down and by the time you have hit number three or four you have lost interest. (…) That is because of academic ranking right. Career progression is linked to your google scholar score.”* |
|  |  | Academic pressure is necessary to publish | *“Most of the time publication is probably motivated by academic career advancement. The more you have, the more you get published.”* |
|  | 3.3 Insufficient time | Publishing is time-consuming | *“Sometimes it takes two years after the trial is finished to actually clean the data. (…) You want to publish it, to present it, when it's finished and you want to publish it. And there is usually a lag of two years before the data is clean and can go in, and I think we need get that shorter.”* |
|  | 3.4 Previous experience influences motivation | There is very little innovation | *“Actually most drugs being developed, the majority are copying. (…) There is very little innovation. Disruptive science is unusual. So I think the challenge to the community is to do more disruptive science.”* |
|  |  | Investigator feels embarrassed when not having published | *“You know I did postgrad research and one of the studies I did not publish was one of the studies attached to my post-grad research and I never finished writing my thesis up. Which is still haunting me.”* |
|  |  | When a pharmaceutical sponsor has a chief investigator that is also an academic, importance of publishing gets better prioritized | *“Leadership plays a big role and I think those who have been in the academic field first, (…) they really push more for promoting the fact that trials needs to be published.”* |
|  | 3.5 Limited incentive or prospect of return | New focus of company prevents publication | *“When I went back to all the studies that I've been PI* (Principal Investigator) *on the last 10-15 years and then actually checked which one were published, and which one (…) didn't get to published. It was not the smaller biotechs. It made me worried. It’s Big Pharma reallocating their resources and not being interested. And actually mentioning that back, or “it's not in our scope anymore”, “We don't have the resources”. It's a multibillion whatever company.”* |
| 4. Inadequate collaboration | 4.1 Insufficient control by regulatory bodies | Regulators cannot enforce their rules | *“How do you enforce it post hoc? Because your regulatory control is all during the trial”* |
|  |  | Contractual obligations cannot be enforced | *“These things* (publishing commitments) *are all usually in the contract. But these are never, ever upheld. You can't bring them to court if they're not publishing. Because there are usually issues why they're not publishing.”* |
|  | 4.2 Different interests between involved parties | Pharmaceutical companies do not put resources into publishing negative drugs | Investigator giving an example of trying to get a study published after three rejections due to too limited data *– “Everyone's moved on. The team that were involved in the trial, they've moved on. There's no prioritization. We wanted a bit more PK but did not get access to that. When we asked the company they said that they don't have the resource to dredge the data and to tabulate it in a certain way. I said that's terrible. That three years after study has been completed, that you can't* (extract more data)*.”* |
|  |  | Company start a later phase trial before publishing phase I trial and therefore do not publish phase I trial | *“For the bigger companies with a big portfolio of agents, they want to publish the ones* (studies) *with the cool data. It is harder to get traction to push them to release the data to investigators. It is even harder to get phase ones, that are just sort of shown as safe dose,* (published) *if the study is in phase two or in combination. It's much harder then, to get those published. It is harder to get the data and then to submit to the journals.”* |
|  |  | New focus of company prevents publication | Investigator giving an example of a study that is not published yet *- “We've had several calls, they're just not interested. They say, “We would love to, but we need to concentrate on the next asset and we don't have the resource to look into”. (…) You know it has to be in agreement that this is going to be published because otherwise why would we put our patients through it.”* |
|  |  | The industry sponsor prevents publishing | *“Sometimes, especially in sponsor trial, it’s the sponsor who is putting roadblocks and don't let that publish. I think academic centers and researchers want this* (study) *to be published. There is a little bit of roadblock if it's a negative study or there is toxicity. Especially in company that that have resources restriction, financially. They want to move on. And they don't put resources in order to complete the clinical study report for example. And they put roadblocks they say “we don't have that?”.”* |
|  |  | There is a problem in publishing when company goes bust | *“There have been situations where actually, literally overnight the company dissolves, run out of funding. (…) You don't get the data and the company you're dealing with is now nonexistent. I don't know what you do in that situation, to be honest. I've had at least two or three in my in my career.”* |
|  |  | The industry runs phase I for profit | Investigator about new commercial research institute *- “They are now a very big stakeholder and running phase ones for the money, it's for profit. And they can open trials very fast and they charge a lot of money. But they're fast and that's what Pharma wants. Now ethically I'm not comfortable with that personally, but Pharma love it. The challenge for me, as an academic investigator, is to be very fast and have a very slick machine operationally so that I can compete with* (name of the new research institute) *and do the trials better.“*  Investigator giving example of not published clinical trial when working with industry partner *–*  *“They blocked it because it didn't look as good as they were making out to their investors, and they didn't want the scrutiny.”* |
|  |  | Trials are not officially closed to prevent publishing the trial | *“They just don't do a formal closeout (…). So they're just moving forward in the development of the drug and they've got the safety data. But if you leave the study open, particularly with some of the studies with multiple arms we've got now, you can end up with one patient somewhere still on the study and it's not closed out.”* |
|  |  | Companies publish data only on trial registry to speed up publication process, but that is insufficient | *“The tricky thing now is that some companies are using the fact that within a a year of declaring trial closure, you have to put the basic summary of data up on clinicaltrials.gov. So it's (*the results) *in the public domain, but that's not the same as a peer reviewed publication because it isn't peer reviewed.”* |
|  |  | Quality of manuscript or analysis is often bad | *“The quality of the papers that do get published, I think, are for the most part useless. They're written by medical writers. The academic authors barely read the papers. And there's no insight whatsoever from at least from an academic perspective.”*  *“I think we have a garbage in garbage out problem.”* |
|  | 4.3 Inadequate collaboration between sponsor & investigators | CRO as a middle party complicates interaction between investigator and sponsor | *“I think the CRO* (Contract Research Organization) *is one of the causes of the problem because it's one layer between you and the Pharma. And that layer is diversified, like across 20 sites. So actually no one takes responsibility.”* |
|  |  | There is a misbalance in the relation between industry and investigator | *“You know if you upset them, they blank you. And they treat you, as though they don't exist anymore and these are very powerful organizations right.”* |
|  |  | A good relationship between investigator and sponsor increases publishing chance | *“Sometimes it boils down to the investigators relationship with the people running the program. Because if there's a tight relationship, it may help sway the company to still consider publication in some form versus programs where the investigators are a bit disconnected from the people leading the trial.”* |
|  |  | Working in a non-academic environment complicates the publication process | *“It depends on how much energy you have. (…) You get busy. And you get busy with patient related care.”* |
|  | 4.4 Information is not shared between sponsor and investigator | Pharmaceutical companies might be cautious to share data to avoid being copied | *“And to be fair to the company executives, you know they are having to protect their company assets. They have no choice. That's what they have to do.”*  *“A new, exciting therapy I presented in this oral presentation. It was big enough to be a plenary oral presentation (…) a couple of years ago. It's still not published. (…) You know there's a lot of deals behind the scenes. And you know it's exciting. Clinical data really needs to be published. But one of the stakeholders don't want it published as it may negatively impact amounts of money they're hoping to raise for their next. And I think to my mind that is ethically wrong.”* |
|  |  | Investigator has to ask for data to push publication | *“We've got one where the CI had copied us other PI's in, and we all backed him up saying; we think this is important, we need to go public, you need to publish the data even if you're abandoning the drug, you still need to put the data in the public domain.”* |
|  |  | Small biotech companies don’t want to share information about promising drugs | *“If the drug works, they want to keep it hidden for as long as possible, so they get sort of bought out.”* |
|  |  | Clinical study report is not readable or imperfect and can therefore not easily be made into a manuscript | *“The only thing you can write a manuscript with, is with the clinical study report. (…) although there is a lot of information there and sometime it misses important pieces.”* |
|  | 4.5 The responsibility for publishing is not properly assigned or taken | The responsibility to publish is shared | *“I think the sponsor has to take the responsibility for it* (the publication of the study)*, but as stakeholders, we also have a responsibility to try and facilitate that and push it along.”* |
|  |  | The chief investigator is mainly responsible for ensuring publication | *“If you signed up on the dotted line as the main person, I think you are the main person for delivering it* (the publication of the study) *(…) and making sure that it* (the study) *goes to the public.”* |
|  |  | Unclear authorship allocation can complicate collaboration | *“In terms of authorship, very often it is something that’s not broached very well. And perhaps that is something that needs to be improved (…) in phase one trials. Authorship should really be determined through contribution, depending on patient accrual, academic contribution and discussions.”* |
|  |  | Pharmaceutical companies are not flexible when they develop a manuscript | *“The Big Pharma’s (…) have very pretty definite ideas of where they want to publish. How they want to publish like clinical on one side, translational on the other side usually. Which to me is not the right way to do.”* |
|  |  | Lack of close out visit prevents a clear start of manuscript writing process | Investigator talking about the occurrence of closing meetings *- “It's not often they’re taking place in many trials. If the patient enrollment to finish my study is finished, it’s the very end. Or it* (the study) *spontaneously naturally disappears, it's gone.”* |
|  |  | If there is no clear lead with a multicenter study, no one has responsibility to ensure publishing | *“They open 30 sites, so none of the PI's* (principal investigators) *are really, you don't own it. So if you do a 3 center study, you're passionate about the drug, you get to own it, even if it's for failure. You know multiple of treated patients and multiple dose levels. Here they open to expansion in 30-40 sites. So there's nobody really in charge of spearheading.”* |
|  | 4.6 Editorial system is not equipped for early phase clinical trials | Positive studies get published more easily | *“I think it's public knowledge that if you have a positive trial, it's going to get published. And it's likely going to get published in a high impact factor journal. So think about those high impact factor journals and that fact. I think there's an unconscious bias.”* |
|  |  | Negative studies are more difficult to publish | *“I think it's becoming more difficult to publish negative trials. It's the usual journals that accept them to be honest, and then these journals, as the impact factor goes up, they also become more fussier to what they accept ultimately. So it changes over time.”* |
|  |  | Phase I trials are less appealing to journals due to relatively poor translational background | *“I think it's getting harder because people are expecting more on the translational stuff. And this translational stuff is weak in negative studies because the industry doesn't always get the money anymore to do all what they have planned on. (..) Even if they have the samples, sometimes they will not push that. And of course, a study with negative results and no translational biomarker is not very appealing for journals.”* |
|  |  | Phase I trials are less appealing to journals due to lack of efficacy as primary endpoint | Investigator giving an example of a study that did not get published *- “We had met the primary, which was not necessarily the efficacy, and had several correlates but there was no efficacy step. We didn't because obviously it wasn't powered to do so. (…) It didn't go to our first journal of interest just because one of the several of the comments of the reviewer “where is my efficacy” (…) I think that there is that component of bias.”* |
|  |  | It is difficult to publish negative studies through peer-review system | *“Sometimes it's very hard to publish a negative study and phase one studies for drugs that have been stopped development. Then it's very difficult to actually get a paper in a peer reviewed journal.”* |
|  |  | The expectations of investigators about their manuscript are too high | *“I think (…) if you haven't got all of the really interesting PK/PD, then it's not likely to be accepted in any significant journal. And some people don't think it's worth their resources or time working it up towards a lesser journal. Then again, that's a problem, isn't it? It still should be published, even if it's in a very low impact factor journal. It should still be in the repository that you can pull on.”* |
|  |  | It is easier for renowned oncologists and big institutions to publish in high impact journals than other investigators | *“I think there's also an unconscious bias of the fact that there are certain renowned oncologist or large institution where they will get in that impact group of journals easier than others. And that's all part of not necessarily consciously, but I think unconsciously, there's a bias there. “* |
|  |  | Reviewers give feedback that is not adapted to early phase trials | *“You'll get feedback from review is saying, well, you shouldn't you have done a X cohort of this, but you can't change the trial when you've done it. It's different with scientific experiments. You can go back and do the additional experiments they're suggesting, but the trial data is the trial data.”* |

## **Table S5. Identified possible solutions to improve the publication of early phase clinical trials**

| **Theme** | **Subtheme** | **Quote** |
| --- | --- | --- |
| A. Suggestions for editors | A.1 Implement new format for difficult to publish papers | *“Journals also want highly cited publications, so no negative studies. They are going to be read, but not going to be cited so much. So why publish this. But perhaps if all journals had a couple of spots in each journal (…) for a section of drugs that are not gonna go. (…) Then everybody in the metric it levels. Because if only one does it, they are publishing a lot and it is bad for the metrics.”*  *“It would be a good initiative to maybe establish recommendations for, you know, negative trials that are not published.“* |
|  | A.2 Journal of negative results | *“You could you even just have a phase one bespoke journal that actually publishes negative data, but more as a repository in a readable format. And if journals are being ranked by their impact, that's not going to be the most attractive. But from a patient point of view, that's really important data to have.”* |
| B. Suggestions for sponsors | B.1 Change how the study data is being made publicly available | *“Something along the line of a short communication, a high impact journal.”*  *“Put together a repository of all completed trials.”*  *“The other aspect is that we should be moving to Open Access data. So that's a different problem with a different solution. I still think if you want to disseminate data, the way to do it is in a peer reviewed journal. But alongside that, we should be putting our data open source so that people can learn from it and interrogate them in their own ways.”*  *“I would much rather just see the companies make the study reports they file with the regulators public. And just create a new publicly accessible database where companies, sponsors, are encouraged to simply submit the study reports they followed with global regulators.”* |
|  | B.2 Increase the sponsor’s sense of responsibility | *“I just think there just needs to be responsibility and accountability on the part of the trial sponsor, who are the IND* (Investigational New Drug) *holders, to ultimately publish.”*  *“At the end of the day, it's the drug company who sponsored the entire endeavor, right? So I do think that they're central to getting the data out there.”* |
| C. Suggestions for investigators | C.1 Emphasize the moral responsibility of investigators | *“I think we also should put in the time and the efforts. Because I personally also sometimes have so many things that perhaps I don’t pursue it* (publishing the study) *so much as I should. So I think we could also use this exercise to remind us of the importance of this. And put the time to it.”*  *“Maybe we as an academic community have to rise up. And demand of the regulators to make this happen.”* |
|  | C.2 Change the procedure for developing and writing of the manuscript | *“We can ask junior investigators or investigators interested in that, to collide the data and write a manuscript as an exercise. And get to know the clinical trials and get to know how to interpret and how to assemble the trial results and interpret the trial results. I think that will be helpful. To get more trials published.”*  *“My experience is that, in general, this* (publication of the study) *is the last topic of discussion. It's like once we've enrolled all the way, the expansion cohorts and everything, then that's when we start discussing manuscripts. And it's a bit of a pain, because often that that point only one piece of data seems to be important and that's the only one that's ever going to be published in extent. So that's where we lose. Because we could probably have done like may be an early dose escalation PK manuscript and then have the efficiency kind of phase Ib/II manuscript, or a second manuscript for that. (…) It would be nice if we could discuss that earlier on in the process.”*  *“So we don't have close out visits. (…) Where are we actually? What's the plan? Who's going to get us the data from the CSR* (clinical study report*)? What do we need from this CSR? And if we haven't got it in the CSR, what PK/PD, etcetera, raw data do we need?”*  *“I'm sponsoring to do a kick off meeting before starting to write the paper, which is very good, but very uncommon. (…) We probably published 20 or 30 studies at per year and I have one or two I kick-off meetings every year.”* |
|  | C.3 Budget more adequate for the publication | *“I think it should be resourced properly. Quite often the trial is resourced, even the archiving is resourced, but actually writing up is not resourced. It's human time, right? Academics do it willingly. But if you had a medical writer or something to create the CSR (clinical study report) and stuff (…) it doesn't require about three to six months of a trained person to write it up. And that should be part of planning to run a study. That* (writing support or time) *should be written in.”* |
| D. Suggestions for regulatory bodies | D.1 Improve enforcement of current standards | *“You have to make it* (publishing study results) *a mandatory requirement. If you make it optional, it's not going to happen. So you have to make your mandate it. That might be unpopular, but it's not unreasonable to be honest.”*  *“From the regulatory perspective, if the health authorities make this mandatory, like they say, “well if you are not doing this, you're not fulfilling your obligations from the health authority perspective”. And then, maybe when they’re presenting a new study to the regulatory agencies, they say, “no, we cannot evaluate your new study because you have pending issues from the prior one”. So this is a way to make it mandatory because contracts cannot.”*  *“In the end, it's for the regulatory agencies. They are the ones who are representing the whole system. (…) So they are the ones who need to control these private companies because they’re using patient data.”* |
|  | D.2 Implement additional regulation | *“If the company goes to the FDA* (Food and Drug Administration) *or the EMA* (European Medicines Agency) *and says “we've done the phase one. Now we come to you to get accelerated approval of Phase two or randomized Phase three.” We should say; “Well, where's the Phase I data?” And it has to be published or we have to give them a timeline for when it has to be published before we're going to sign off on this thing. So I think that they could enforce that.”*  *“You have an obligation to our patients and the Institute has the same obligation. So it's not that strange to put it in a contract.”* |
|  | D.3 Use financial repercussion | *“It may be simpler initially, just to make it a mandate* (to publish study results), *and see what happens. And if people don't do it and they are serial offenders, then start talking about (…) fiscal repercussions.”* |
| E. Suggestions for society | E.1 Use reputation as an incentive | *“An initial question that everyone can ask, as investigators, of the company (…) “what's your track record publishing more positive and negative trials with investigators? What's your track record?” Because that can guide you and the expectation would be that we would like to publish it. So you know the companies are prepared to share that data.”*  *“If you are going to put it in a big contract, nobody is going to go after big pharma company. So I don’t think that works. I think it should be more of a reputation aspect or something you would want to show off. A certificate that we pass after an audit, (…) and you can actually see where the money is going and we do this accreditation. And this is a voluntary thing to do. Because then, of course, if you are not doing it (the accreditation) then you are the one that is strange. So somewhere you need to show this data. (for example) I have performed, in the last year, I have performed 80 studies, I have finished 60 studies, I have published 20. If this is a metric that you show. The pressure in itself it would be.. If you are quantifying the metric and people are asking for the metric. I think that would be much more useful than putting it in any kind of contract. It is just reputation and building reputation against that metric. And showing investigators when they come and show you their trial (… ) they (the sponsors) show you their trial metric of publications.”*  *“I think we can utilize social media …. So I think the industry partner having made to be morally obligated is good. I think social media is good nowadays, big pharma would care about the image.”* |
|  | E.2 Use pressure from organizations or society | *“I think the societies can help because it's an aggregate of people who practice in different environment and if a big society pushed to say we're not going to do more studies unless the companies agree to publish whatever happens, then maybe it'll become more commonplace.”*  *“Perhaps solutions to this would be patient advocacy groups really putting pressure on companies to actually publish data.”*  *“The patient advocate voice is very important and well heard. (…) They could put pressure on the regulators that you should have this mandate to publish.“*  *“Maybe we as an academic community have to rise up. And demand of the regulars to make this happen.”*  *“I mean the ethics committees (…) once they've given approval of the study, I don't know what leverage they would have. Unless they said we'll only approve a study if you are going to publish within X of thing. But it's not going to have any leverage at that point, is it? Only if somebody then goes back to the same ethics committee for another study.”* |

## **Table S6. SRQR checklist**

| **Standards for Reporting Qualitative Research (SRQR)*** |  | |  |
| --- | --- | --- | --- |
|  |  | |  |
|  | **Page/line no(s).** | |  |
| **Title and abstract** | |  | |
| **Title** - Concise description of the nature and topic of the study Identifying the study as qualitative or indicating the approach (e.g., ethnography, grounded theory) or data collection methods (e.g., interview, focus group) is recommended | 1 | |  |
| **Abstract** - Summary of key elements of the study using the abstract format of the intended publication; typically includes background, purpose, methods, results, and conclusions | 1 | |  |
|  |  | |  |
| **Introduction** | |  | |
| **Problem formulation** - Description and significance of the problem/phenomenon studied; review of relevant theory and empirical work; problem statement | 2 | |  |
| **Purpose or research questio**n - Purpose of the study and specific objectives or questions | 2 | |  |
|  |  | |  |
| **Methods** | |  | |
| **Qualitative approach and research paradigm** - Qualitative approach (e.g., ethnography, grounded theory, case study, phenomenology, narrative research) and guiding theory if appropriate; identifying the research paradigm (e.g., postpositivist, constructivist/ interpretivist) is also recommended; rationale** | 2 | |  |
| **Researcher characteristics and reflexivity** - Researchers’ characteristics that may influence the research, including personal attributes, qualifications/experience, relationship with participants, assumptions, and/or presuppositions; potential or actual interaction between researchers’ characteristics and the research questions, approach, methods, results, and/or transferability | 2 | |  |
| **Context** - Setting/site and salient contextual factors; rationale** | 2 | |  |
| **Sampling strategy** - How and why research participants, documents, or events were selected; criteria for deciding when no further sampling was necessary (e.g., sampling saturation); rationale** | 2 | |  |
| **Ethical issues pertaining to human subjects** - Documentation of approval by an appropriate ethics review board and participant consent, or explanation for lack thereof; other confidentiality and data security issues | 11 | |  |
| **Data collection methods** - Types of data collected; details of data collection procedures including (as appropriate) start and stop dates of data collection and analysis, iterative process, triangulation of sources/methods, and modification of procedures in response to evolving study findings; rationale** | 2–3 | |  |
| **Data collection instruments and technologies** - Description of instruments (e.g., interview guides, questionnaires) and devices (e.g., audio recorders) used for data collection; if/how the instrument(s) changed over the course of the study | 2–3 | |  |
| **Units of study** - Number and relevant characteristics of participants, documents, or events included in the study; level of participation (could be reported in results) | 2–3 | |  |
| **Data processing** - Methods for processing data prior to and during analysis, including transcription, data entry, data management and security, verification of data integrity, data coding, and anonymization/de-identification of excerpts | 2–3 | |  |
| **Data analysis** - Process by which inferences, themes, etc., were identified and developed, including the researchers involved in data analysis; usually references a specific paradigm or approach; rationale** | 3 | |  |
| **Techniques to enhance trustworthiness** - Techniques to enhance trustworthiness and credibility of data analysis (e.g., member checking, audit trail, triangulation); rationale** | 2 | |  |
|  |  | |  |
| **Results/findings** | |  | |
| **Synthesis and interpretation** - Main findings (e.g., interpretations, inferences, and themes); might include development of a theory or model, or integration with prior research or theory | 3–7 | |  |
| **Links to empirical data** - Evidence (e.g., quotes, field notes, text excerpts, photographs) to substantiate analytic findings | 3–7 + Supplementary tables | |  |
|  |  | |  |
| **Discussion** | |  | |
| **Integration with prior work, implications, transferability, and contribution(s) to the field -** Short summary of main findings; explanation of how findings and conclusions connect to, support, elaborate on, or challenge conclusions of earlier scholarship; discussion of scope of application/generalizability; identification of unique contribution(s) to scholarship in a discipline or field | 7–8 | |  |
| **Limitations** - Trustworthiness and limitations of findings | 7–8 | |  |
|  |  | |  |
| **Other** | |  | |
| **Conflicts of interest** - Potential sources of influence or perceived influence on study conduct and conclusions; how these were managed | 8–10 | |  |
| **Funding** - Sources of funding and other support; role of funders in data collection, interpretation, and reporting | 8 | |  |
|  |  | |  |
| *The authors created the SRQR by searching the literature to identify guidelines, reporting standards, and critical appraisal criteria for qualitative research; reviewing the reference lists of retrieved sources; and contacting experts to gain feedback. The SRQR aims to improve the transparency of all aspects of qualitative research by providing clear standards for reporting qualitative research. |  | |  |
|  |  | |  |
| **The rationale should briefly discuss the justification for choosing that theory, approach, method, or technique rather than other options available, the assumptions and limitations implicit in those choices, and how those choices influence study conclusions and transferability. As appropriate, the rationale for several items might be discussed together. |  | |  |
|  |  | |  |
| **Reference:** |  | |  |
| O'Brien BC, Harris IB, Beckman TJ, Reed DA, Cook DA. **Standards for reporting qualitative research: a synthesis of recommendations.** *Academic Medicine*, Vol. 89, No. 9 / Sept 2014 DOI: 10.1097/ACM.0000000000000388 |  | |  |
